# Omega-3 fatty acids in high-risk cardiovascular patients: a meta-analysis of randomized controlled trials

**DOI:** 10.1186/1471-2261-10-24

**Published:** 2010-06-03

**Authors:** Kristian B Filion, Fouad El Khoury, Michael Bielinski, Ian Schiller, Nandini Dendukuri, James M Brophy

**Affiliations:** 1 Department of Medicine, McGill University, Montreal, Quebec, Canada; 2 Department of Epidemiology, Biostatistics, and Occupational Health, McGill University, Montreal, Quebec, Canada; 3 Technology Assessment Unit, McGill University Health Center, Montreal, Quebec, Canada

## Abstract

**Background:**

Multiple randomized controlled trials (RCTs) have examined the cardiovascular effects of omega-3 fatty acids and have provided unexplained conflicting results. A meta-analysis of these RCTs to estimate efficacy and safety and potential sources of heterogeneity may be helpful.

**Methods:**

The Cochrane library, MEDLINE, and EMBASE were systematically searched to identify all interventional trials of omega-3 fatty acids compared to placebo or usual diet in high-risk cardiovascular patients. The primary outcome was all-cause mortality and secondary outcomes were coronary restenosis following percutaneous coronary intervention and safety. Meta-analyses were carried out using Bayesian random-effects models, and heterogeneity was examined using meta-regression.

**Results:**

A total of 29 RCTs (n = 35,144) met our inclusion criteria, with 25 reporting mortality and 14 reporting restenosis. Omega-3 fatty acids were not associated with a statistically significant decreased mortality (relative risk [RR] = 0.88, 95% Credible Interval [CrI] = 0.64, 1.03) or with restenosis prevention (RR = 0.89, 95% CrI = 0.72, 1.06), though the probability of some benefit remains high (0.93 and 0.90, respectively). However in meta-regressions, there was a >90% probability that larger studies and those with longer follow-up were associated with smaller benefits. No serious safety issues were identified.

**Conclusions:**

Although not reaching conventional statistical significance, the evidence to date suggests that omega-3 fatty acids may result in a modest reduction in mortality and restenosis. However, caution must be exercised in interpreting these benefits as results were attenuated in higher quality studies, suggesting that bias may be at least partially responsible. Additional high quality studies are required to clarify the role of omega-3 fatty acid supplementation for the secondary prevention of cardiovascular disease.

## Background

Interest in the therapeutic value of fish oils began in the 1970's following the observation of a low incidence of cardiovascular disease in Greenland Inuits [1-3]. Subsequently, several studies identified eicosapentaenoic acid (EPA) and docosahexaenoic acid (DHA), two major long chain n-3 polyunsaturated fatty acids (n-3 PUFAs), as the putative protective constituents [4-10]. The role of α-linolenic acid (ALA), a shorter chain omega-3, is still being debated [11]. Although the exact cardio-protective mechanisms of omega-3 fatty acids are unknown, it has been hypothesized that a reduction of arrhythmias, heart rate, ischemia/reperfusion-induced injury, serum triglyceride levels, inflammation, or improved endothelial function may be involved [12-17]. Multiple studies, reviews, and meta-analyses have been conducted in order to elucidate the effect of omega-3 fatty acids on cardiovascular outcomes, but controversy prevails with both positive [18] and negative conclusions [19,20].

Omega-3 fatty acids are inexpensive compounds with an apparent favorable risk profile including a low propensity for drug interaction. Moreover, there have been several recent publications of randomized trials, and we therefore decided to perform an updated systematic review and meta-analysis of their cardiovascular efficacy. We have, unlike previous analyses [18,19], restricted our systematic review to high risk patients, the group most likely to benefit from their use, and have explicitly investigated potential sources of heterogeneity between these trials.

## Methods

### Literature Search

We searched MEDLINE, the Cochrane Library, and EMBASE without language restrictions for original research articles, systematic reviews, and meta-analyses to identify all available literature on omega-3 fatty acids and cardiovascular disease published from January 1966 through September 2008. We combined search terms for omega-3 fatty acids ("omega-3 fatty acids" OR "fish oil" OR "marine oil" OR "dietary therapy") with those for mortality or cardiovascular disease ("mortality" OR "cardiovascular disease" OR "heart disease" OR "CAD" OR "MI" OR "UA" OR "coronary angiography" OR "coronary restenosis"). References of relevant articles were hand-searched for additional studies.

Inclusion criteria required each study to 1) be a comparative randomized trial involving human participants with an active treatment arm using omega-3 fatty acids with usual diet; 2) involve a high-risk population with known cardiovascular disease or diabetes; and 3) report at least 1 of the following 2 outcomes: total mortality or coronary artery restenosis following angioplasty. We excluded non-randomized studies, those involving children or animals, and studies in which the omega-3/fish supplement dosage was unspecified. We also excluded studies in which patients were randomized to dietary advice that included a non-quantifiable intervention of simply increasing fish consumption [21]. The Quality of Reporting of Meta-analyses of Randomized Controlled Trials (QUORUM) guidelines were followed throughout this meta-analysis [22] [See Additional file 1 - QUORUM checklist].

### Data Extraction

One author performed the literature search, and data extraction was independently conducted by at least two individuals. The following information was extracted: publication details, timing of study, duration of follow-up, randomization method, blinding (of participants, investigators, and outcome assessors), omega-3 dosage, dropouts, mean age of participants, primary outcome (total mortality), secondary cardiovascular outcomes (restenosis, sudden death, cardiac death, non-fatal myocardial infarction (MI), congestive heart failure (CHF), arrhythmias, implantable cardioverter defibrillator (ICD) shocks, and stroke), the proportion of patients who discontinued treatment, side effects (gastro-intestinal (GI) side effects, bleeding, and malignancies), and adherence. Any disagreements in the collected data was resolved by consensus or, when necessary, upon consultation with a third reviewer. The authors of the original publications were contacted to obtain missing data and resolve ambiguities (n = 16), although we made no attempt to contact authors of articles published before 1995.

Quality assessment of the individual trials was performed using the Jadad scale [23] [See Additional file 2-Quality assessment of included trials].

### Statistical Analysis

To estimate the risk of all-cause mortality, data were analyzed according to the intention-to-treat principal; the denominator was the number of participants randomized to each group, and the numerator was the number of deaths reported during the follow-up period. In our restenosis analysis, the denominator was the number of participants undergoing follow-up coronary angiography, and the numerator was those with restenosis. In most studies, restenosis was defined as the loss of luminal diameter of at least 50% [24-29]. One study defined it as a loss of 70% [30], and 2 studies defined coronary restenosis as at least 50% stenosis at follow-up [31,32]. Three trials used multiple definitions, including loss of luminal diameter of at least 50% and at least 50% stenosis at follow-up [33-35]. The remaining two studies used unique restenosis definitions; one used a panel of blinded cardiologists to assess changes in progression and regression of CAD [36], and the other defined restenosis as lumens with greater than 20% obstruction [37]. Where possible, cardiovascular event and safety data were analyzed using an intention-to-treat approach. However, many restenosis studies presented data only for those who underwent follow-up coronary angiography.

For each study, we estimated the risk ratio (RR) comparing intervention and control groups. For studies with zero outcomes in either group, we added 0.5 to all cells of the 2-by-2 table. For the primary outcomes of mortality and main secondary analysis of restenosis, we fit meta-regression models to investigate if study-level covariates explained any of the heterogeneity in RRs across studies. We estimated the median and corresponding 95% credible interval (CrI), the Bayesian analogue for confidence intervals, for each coefficient in these models. In addition, we also estimated the probability that the coefficient was greater than 0. The covariates used in the meta-regression were median follow-up time (months), sample size, high dosage of the intervention, high quality (on the Jadad scale), high adherence rate, and high percentage of previous MI. Cut-offs for defining dichotomous covariates were determined by the median value across studies. For the all-cause mortality meta-analysis, subgroups were defined by sample size (>322 patients), study quality (>3), dosage of omega-3 (>3.3 g/day), adherence (>84%), and history of previous MI (>50%). For the restenosis meta-analysis, subgroups were defined by study quality (>3), sample size (>233 patients), dosage of omega-3 (>5.04 g/day), adherence (>85%), and previous MI (>48%).

We carried out meta-analyses to pool RRs across all studies [38]. Separate models were created for each of the primary and secondary outcomes and for safety data. When there was greater than 90% probability that a covariate was associated with the relative risk (i.e., >90% probability that the meta-regression coefficient was different from 0), we carried out separate meta-analyses within subgroups defined by the covariate. Funnel plots were employed to assess publication bias.

All analyses were carried out using WinBUGS and R software programs. Low information prior distributions (mean 0 and a standard deviation of 100) were used for all parameters. The between-study standard deviation in the log (RR) was assumed to follow a uniform distribution over the range from 0 to 5.

## Results

### Literature Search

**Figure 1:**
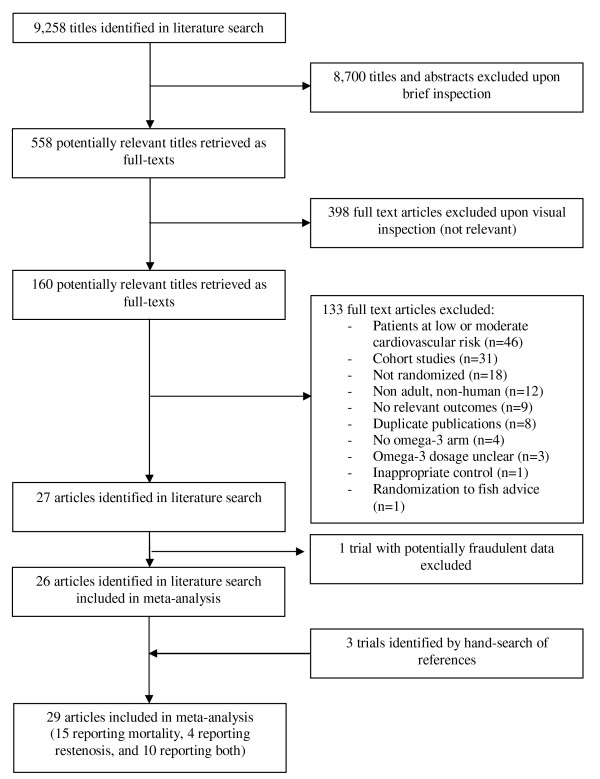
**Flow diagram of selection process of randomized controlled trials included in meta-analysis**.

Our literature search identified 9,258 titles, of which 558 articles were considered potentially relevant (Figure 1). The full texts of these articles were retrieved, and 27 articles fulfilled our inclusion criteria [24-37,39-51]. One trial [51] was then excluded due to concerns regarding the authenticity of its data [52-54]. Three additional trials were identified through our hand-search of relevant studies [55-57], resulting in a total of 29 included trials. These trials involved a total of 35,144 patients. There were 25 articles that reported mortality [24-26,29,31,33-37,39-50,55-57] and 14 that examined coronary restenosis [25-37,42]. The trials reporting mortality randomized 34,501 patients (17,276 omega-3 patients and 17,190 controls), and the restenosis trials randomized 3,553 patients to omega-3 fatty acids (n = 1,817) or control (n = 1,736).

### Study and Patient Characteristics

Although performed in a multitude of different countries, the trials were fairly homogeneous with respect to patient and study characteristics [See Additional file 3 - study and patient characteristics]. All mortality trials were conducted in high-risk cardiovascular patients: 4 were conducted in post-MI patients [21,24,43,44], 3 involved patients with ICDs [47-49], and 1 involved diabetic patients [41]. The remaining 16 trials consisted of a mixture of other high-risk cardiovascular patients. Most trials were conducted in middle aged or older patients, and the majority of patients were men. Trials were generally double-blind, with either placebo or inactive oil as control, although some were open-label with control being usual diet [28,44]. Thirteen mortality trials and 2 restenosis trials had follow-up ≥ 12 months. In the mortality trials, omega-3 was provided in the form of EPA, DHA, DPA, or ALA, with dosages ranging from 0.9 g/day to 6.9 g/day. In the restenosis trials, omega-3 consisted of EPA or DHA, with daily dosages ranging from 3 g/day to 6.9 g/day. Follow-up varied from 1 to 55 months with a median 6 months for restenosis studies and 12 months for mortality studies.

### All-Cause Mortality

**Figure 2:**
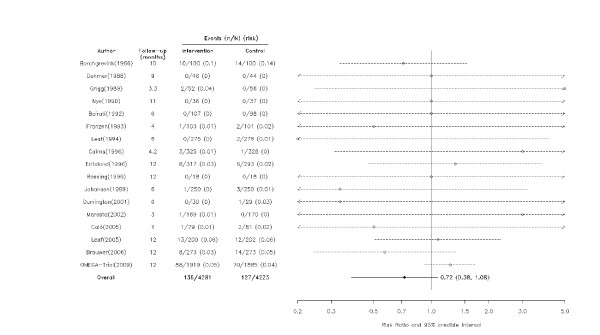
**Effect of omega-3 fatty acids on all-cause mortality among studies with follow-up ≤ 12 months**. Arrows indicate that the lower limit of the credible interval for the relative risk was less than 0.2 or the upper limit exceeded 5.0.

**Figure 3:**
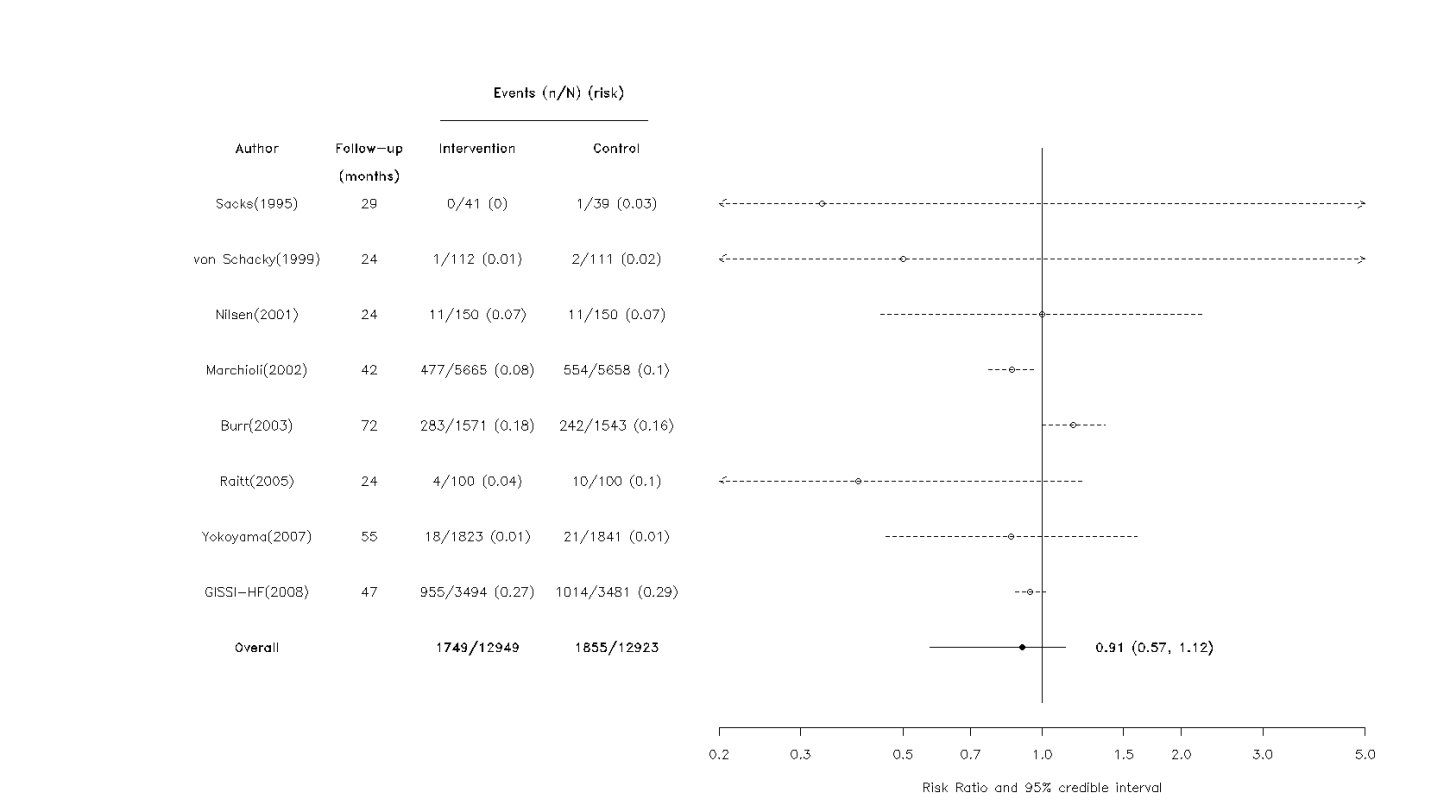
**Effect of omega-3 fatty acids on all-cause mortality among studies with follow-up > 12 months**. Arrows indicate that the lower limit of the credible interval for the relative risk was less than 0.2 or the upper limit exceeded 5.0.

A total of 3,867 deaths (1,885 in omega-3 patients and 1,982 in controls) occurred in these trials. When data were pooled across all studies, omega-3 fatty acids were not associated with a reduction in all cause-mortality (RR = 0.88, 95% CrI = 0.64, 1.03), though the probability of some benefit (RR<1) remains high (0.93). The majority of the studies were inconclusive but one individual study with a follow-up greater than 12 months did show a statistically significant beneficial effect of omega-3 fatty acids (Figures 2, 3).

**Figure 4:**
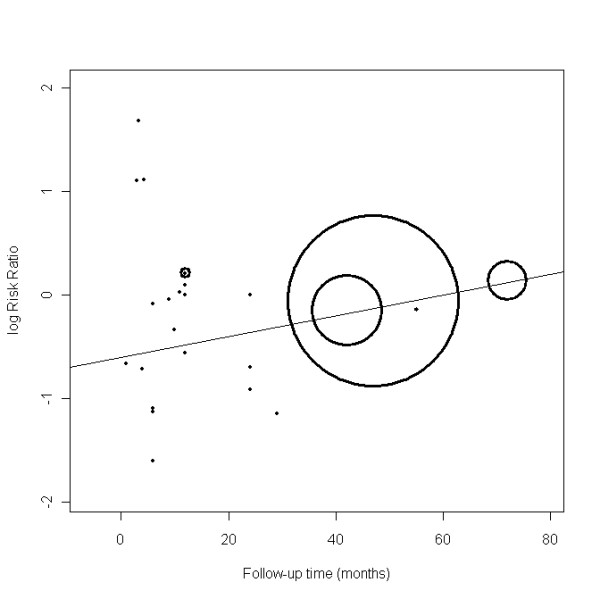
**Scatter plot of the change in log (risk ratio) for all-cause mortality with median follow-up time**. Weights in the scatter plot are proportional to the inverse of the variance of each study's log (risk ratio).

The meta-regression models suggested with a greater than 90% probability that larger studies and those with longer follow-up were associated with decreasing health benefits (Table 1). Specifically, there was a 97% probability that the effect of omega-3 fatty acids was smaller in the 12 larger trials (RR = 0.95, 95% CrI = 0.76, 1.13) than in the 13 trials with small sample sizes (RR = 0.47, 95% CrI = 0.18, 0.83). Similarly, there was a 99% probability that those with follow-up greater than 12 months (RR = 0.91, 95% CrI = 0.57, 1.12) had attenuated benefits compared with those with follow-up ≤ 12 months (RR = 0.72, 95% CrI = 0.38, 1.08) (Figures 2, 3 and 4). Dosage, study quality, adherence, and history of previous MI did not appear to influence the results.

**Table 1:** Results of univariate meta-regression models relating mortality and restenosis regression coefficients to study-level covariates*.

	Mortality		Restenosis	
	Median Regression Coefficient (95% CrI)	Probability that the Regression Co-efficient>0	Median Regression Coefficient (95% CrI)	Probability that the Regression Co-efficient>0
Follow-up time (months)	0.009 (0.003, 0.019)	0.992	0.00 (-0.03, 0.03)	0.4821
High dosage	-0.48 (-1.123, 0.141)	0.059	0.05 (-0.39, 0.45)	0.6301
High quality	-0.247 (-0.837, 0.39)	0.208	0.22 (-0.15, 0.65)	0.8909
High adherence rate	0.16 (-0.331, 0.529)	0.81	-0.29 (-1.16, 0.58)	0.1909
High percentage of previous MI	0.11 (-0.493, 0.434)	0.712	0.03 (-0.43, 0.54)	0.6155
Log (sample size)	0.16 (-0.005, 0.402)	0.971	0.19 (-0.07, 0.46)	0.94

Abbreviations: CrI: credible interval; MI: myocardial infarction.

*High dosage, quality, adherence, and percentage of previous MI were defined using the medians of all studies.

We also carried out a sensitivity analysis by excluding the sole study using ALA [24], which did not change the overall results.

### Coronary Restenosis

**Figure 5:**
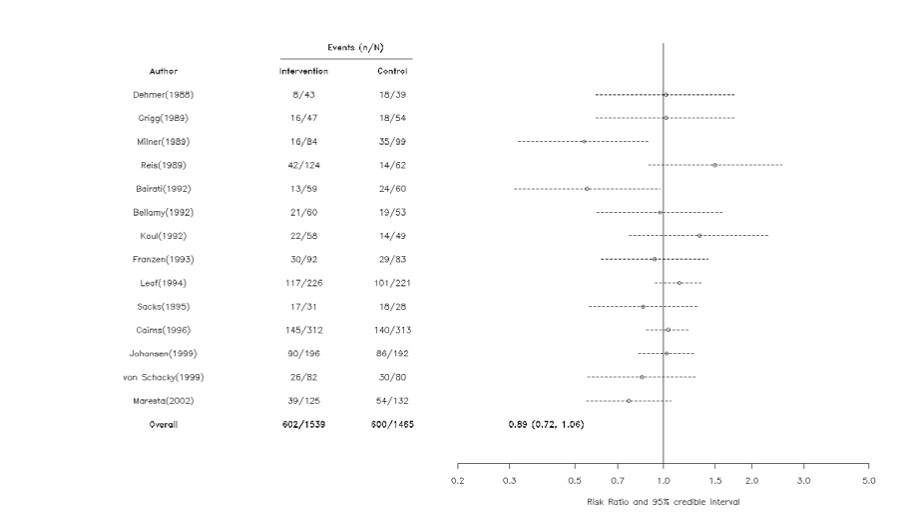
**Effect of omega-3 fatty acids on coronary restenosis**. Arrows indicate that the lower limit of the credible interval for the relative risk was less than 0.2 or the upper limit exceeded 5.0. Although 3,553 patients were randomized to omega-3 fatty acids (n = 1,817) or control (n = 1,736), only 3,004 patients (1,539 and 1,465, respectively) were included in the analyses. Patients who did not undergo follow-up angiogram were generally excluded from the restenosis analyses.

In 14 studies that assessed omega-3 fatty acids following a percutaneous coronary intervention (PCI), these agents were not statistically associated with the risk of restenosis (RR = 0.89, 95% CrI = 0.72, 1.05)(Figure 5) but again there was a moderate probability of some benefit (probability RR < 1 = 0.90). Subgroup analysis suggested that this result was unaltered by dosage, follow-up, or adherence (Table 1) but there was a 94% probability that the effect of omega-3 fatty acids on restenosis was attenuated in larger RCTs (RR = 0.94, 95% CrI = 0.66, 1.26) compared with smaller ones (RR = 0.82, 95% CrI = 0.56, 1.17) and a 89% probability that higher quality trials also exhibited attenuated results.

### Other Cardiovascular Events

Although the point estimates suggest that omega-3 fatty acids may reduce other cardiovascular events, including cardiac death, sudden death, non-fatal MI, CHF, arrhythmias, and ICD shocks, the wide CrIs prevent definitive conclusions from being drawn [See Additional file 4 - secondary cardiovascular outcomes].

### Safety

Few studies reported side effect and safety data (Table 2). Patients randomized to omega-3 fatty acids did have an increased risk of GI side-effects (RR = 1.29, 95% CrI = 1.02, 1.61). However, the risk of treatment discontinuation was similar between groups (RR = 1.23, 95% CrI = 0.87, 1.68), suggesting that these side-effects were relatively minor. There was no increased risk of malignancies (RR = 1.02, 95% CrI = 0.73, 1.25) or bleeding (RR = 0.85, 95% CrI = 0.52, 1.27), but wide 95% CrI again prevent definitive conclusions from being drawn regarding these outcomes.

**Table 2:** Randomized controlled trials examining the safety of omega-3 fatty acids.

	Number of Patients		Discontinued Treatment		GI Side Effects		Bleeding		Cancer	
**Study**	**Fish Oil**	**Ctrl**	**Fish Oil**	**Ctrl**	**Fish Oil**	**Ctrl**	**Fish Oil**	**Ctrl**	**Fish Oil**	**Ctrl**

Borchgrevink [24]	100	100	9	9	7	7	NR	NR	0^††^	1^††^
Dehmer [31]	43	39	0	NR	7	3	3	1	NR	NR
Grigg [29]	52	56	2	NR	2	1	0	0	NR	NR
Milner [32]	95	99	11	0	24	NR	0	NR	NR	NR
Reis [30] *	124*	51*	NR	NR	59	11	4	0	NR	NR
Nye [39]	36	37	NR	NR	NR	NR	NR	NR	NR	NR
Bairati [33]	59	60	NR	NR	NR	NR	0	0	NR	NR
Bellamy [28]^†^	60	60^†^	3	0^‡^	5	0	NR	NR	NR	NR
Kaul [27]	58	49	NR	NR	2	0	0	0	NR	NR
Franzen [34]^§^	103^§^	101^§^	6	7	14	10	0	0	NR	NR
Leaf [25]	226	221	2	7	16	18	7	7	NR	NR
Sacks [37]^¶^	41^¶^	39^¶^	6	1	NR	NR	0	0	NR	NR
Cairns [26]	325	328	NR	NR	122	101	17	38	NR	NR
Eritsland [40]	317	293	41	25	40	30	5	4	NR	NR
Rossing [41]	18	18	4	3	3	1	0	0	1	1
Johansen [42]^||^	196^||^	192^||^	NR	NR	3	2	NR	NR	NR	NR
von Schacky [36]	111	112	14	15	4	3	0	0	0	0
Nilsen [43]	150	150	NR	NR	NR	NR	NR	NR	2**	0**
Durrington [56]	30	29	1	1	1	1	0	0	0	0
Marchioli [44]	5666	5658	1616	687^‡^	278	NR	NR	NR	142	134
Maresta [35]^††^	146^††^	141^††^	NR	NR	2	2	6	3	NR	NR
Burr [45]	1571	1543	NR	NR	NR	NR	NR	NR	51**	47**
Calò [46]	79	81	NR	NR	NR	NR	1	1	NR	NR
Leaf [47]	200	202	73	69	NR	NR	NR	NR	NR	NR
Raitt [48]	100	100	17	26	11	12	NR	NR	3	4
Brouwer [49]	273	273	23	18	17	12	NR	NR	4	4
Yokoyama [50]^‡‡^	9326	9319	1087	673	352	155	105	60	242	218
GISSI-HF[55]	3494	3481	102	104	96	92	NR	NR	NR	NR
OMEGA-Trial [57]	1919	1885	NR	NR	NR	NR	35	34	NR	NR
**Relative Risk (95% CrI)**			**1.23 (0.87, 1.68)**		**1.29 (1.02, 1.61)**		**0.85 (0.52, 1.27)**		**1.02 (0.73, 1.25)**	

Abbreviations: Ctrl = control; GI = gastrointestinal; NR = not reported.

* Reis et al. [30] randomized 222 patients, 204 of which underwent angioplasty. Of these patients, 14 patients had unsuccessful angioplasties and 4 other patients underwent bypass surgery following their angioplasty. These 18 patients were excluded from further study. The remaining 186 patients were included in the study, including 124 patients in the fish oil group (60 randomized to ethyl ester fish oil, 64 randomized to triglyceride fish oil) and 62 randomized to placebo. Clinical event data were available for all patients however 11 patients in the placebo group did not provide side effect data. Thus, side effect analyses are based on 124 fish oil patients and 51 placebo patients.

^† ^The control group involved a total of 60 patients, 53 of which underwent follow-up angiography and 7 of which who did not. Consequently, the sample size of the control group is 53 patients for baseline and restenosis data but 60 patients for all other analyses.

^‡^The study by Bellamy et al. [28] did not involve the use of placebo and thus, treatment discontinuation data were not presented for the control group. The study by Marchioli et al. [44] was a factorial design in which the control group was comprised of 2,830 patients randomized to vitamin E and 2,828 patients randomized to control. Discontinuation data were not available for those randomized to control.

^§^In the study by Franzen and colleagues [34], 204 patients were randomized to fish oil (n = 103) or placebo (n = 101). However, only 175 patients underwent angiographic follow-up (92 fish oil and 83 placebo). Consequently, the sample size for restenosis should be a total of 175 patients whereas the sample size for all other analyses should be 204.

^¶^Sacks and colleagues [37] randomized 80 patients to fish oil (n = 41) or control (n = 39). A total of 21 patients were excluded from the study prior to undergoing their follow-up angiogram. Although clinical events were assessed in all 80 randomized patients, restenosis was only assessed in 59 patients (31 fish oil, 28 controls).

^||^Johansen and colleagues [42] randomized a total of 500 patients to omega-3 fatty acid (n = 250) or placebo (n = 250). Of these patients, 5 were disqualified before their angioplasty (4 omega-3 patients and 1 placebo patient), 103 were disqualified during their angioplasty (n = 49 and 54, respectively), and 4 patients were excluded because they died prior to their follow-up angiogram (1 omega-3 patient and 3 patients randomized to placebo). There were therefore 196 patients randomized to omega-3 fatty acids and 192 patients randomized to placebo who were ultimately included in the study. The cardiac death analyses are based on 197 patients randomized to omega-3 fatty acids and 195 patients randomized to placebo whereas all other analyses are based on 196 and 192 patients, respectively.

**Data obtained from previous Cochrane review [19].

^†† ^Maresta et al. [35] randomized 339 patients (169 to fish oil, 170 to placebo). Of these patients, 52 were disqualified before or at their angioplasty, leaving 287 patients (146 fish oil, 141 placebo). Side effect and clinical event data are based on these patients. An additional 23 patients were unevaluable drop-outs, leaving 264 patients (129 fish oil, 135 placebo) with 6-month angiograms available, and 7 additional patients were excluded because quantitative coronary angiography was not possible, resulting in a total of 257 patients (125 fish oil, 132 placebo) being included in the restenosis analyses.

^‡‡^The JELIS Trial [50] (conducted by Yokoyama) was performed on a population with low, moderate and high risk cardiovascular patients. Due to the lack of data, cardiovascular instead of all-cause mortality was used for the secondary prevention group. Demographic and cardiovascular outcome data are presented for the high-risk group (n = 3,664) whereas safety data are presented for the entire study population (n = 18,645). The JELIS cardiovascular data were included as part of our pooled analysis. However, due to the heterogeneity of the entire study population, data from the JELIS trial were not included in our pooled analysis of safety data.

Funnel plots did not provide any clear indication of publication bias (data not shown).

## Discussion

Our systematic review and meta-analysis assessed the effect of omega-3 fatty acids on all-cause mortality and coronary artery restenosis following PCI among high-risk cardiovascular patients. Omega-3 fatty acids were not associated with a statistically significant reduction in all-cause mortality or restenosis but the probability of a modest benefit remains considerable. Omega-3 fatty acids also had generally favorable effects on other cardiovascular outcomes, but definitive conclusions are not forthcoming due to the small number of studies that reported these outcomes. Unfortunately, the majority of trials did not systematically record adherence and side effects. Nevertheless, the available data suggest a favorable side-effect profile.

Our meta-regression identified some important sources of heterogeneity among mortality trials, including trial size and follow-up time. Specifically, larger and longer trials had smaller mortality benefits, suggesting that the overall observed benefit may be at least partially inflated due to bias. Similarly, restenosis benefits were smaller in larger, better quality trials. These findings temper our enthusiasm for this intervention despite a relatively favorable risk profile. Definitive results about the efficacy and safety of omega-3 fatty acid supplementation will benefit from the results of currently ongoing clinical trials, including ORIGIN [58] and ASCEND [59].

The effect of omega-3 fatty acids has been examined in previous systematic reviews and meta-analyses. However, these earlier meta-analyses [19,60,61] were not limited to high-risk cardiovascular patients, included a scientifically questionable study [51], did not have access to the most recently published large studies of 15,000 high-risk cardiovascular patients [47-50,55,57], and importantly, did not explicitly investigate potential sources of heterogeneity which permits a more nuanced interpretation of the totality of evidence. Our results are similar to previous focused meta-analysis examining restenosis [62] and ICD shocks [61,63]. However, our credible intervals are slightly wider as our Bayesian methods account for uncertainty in the between-study variability.

### Strengths and Limitations

Our systematic review and meta-analysis has a number of strengths. First, it provides a complete and comprehensive review of the current state of the omega-3 fatty acid literature. In several cases, the published data of this systematic review have been complimented by additional data furnished by the principal investigators of the original studies. Second, our Bayesian models, unlike their frequentist counterparts, allow for the calculation of probabilities and therefore have a more intuitive and informative interpretation. Third, our systematic review and meta-analysis was conducted according to a pre-specified protocol, including pre-specified subgroup analyses, and without language restrictions. Fourth, we have addressed not only the efficacy but also the safety of omega-3 fatty acids. Finally, although previous reports have discussed the role of heterogeneity in the literature [64], we examined the sources of heterogeneity analytically. Consequently, we have provided a thorough and methodologically rigorous synthesis of the available evidence thereby facilitating informed decision making.

Nevertheless, our systematic review and meta-analysis does have potential limitations. First, as is true with all systematic review and meta-analyses, our study may be affected by publication bias, although we did not find evidence of its occurrence. Second, there was some heterogeneity in study design, including in dosage of omega-3 fatty acid used and patient populations. In particular, there is much uncertainty regarding the potency and purity of over-the-counter formulations while the proprietary formulation is both expensive and has been infrequently used in the clinical trials. Moreover, the control groups were exposed to varying amounts of fish oils according to national dietary habits, and we could not account for this variability. Our random-effects models attempt to account for between-study variability, and the effects of this heterogeneity were examined in our meta-regression models. Third, safety data were not reported in all studies. Fourth, due to the fish odor of omega-3 fatty acid supplements, complete blinding of fish oil studies may not be feasible. This imperfect blinding was not considered in our quality assessment. Fifth, most restenosis studies only presented data among those who completed their follow-up angiogram. Consequently, restenosis data were generally analyzed using a modified intention-to-treat, which may result in biased results. Finally, due to the lack of individual-level data, we were not able to estimate the change in risk of mortality or cardiovascular outcomes over time. We therefore assumed that the risk of the outcome remained the same across the duration of each study and that any censoring was random. Availability of individual-level data would also have allowed us to estimate the effect of patient-level covariates and to examine which subgroups may derive the greatest benefit from the use of these agents.

## Conclusions

This meta-analysis demonstrates that omega-3 fatty acid supplementation may modestly reduce all-cause mortality and restenosis when used as secondary prevention. However, the mortality benefit was attenuated in larger RCTs and those with longer follow-up, and the restenosis benefits were similarly reduced in larger RCTs. These results suggest that bias may at least partially explain the observed benefit. Also, many studies had incomplete information on other cardiac endpoints and adverse events. Further ongoing studies with sufficient sample size, standardized dosing, and adequate follow-up duration are required to clarify the role of omega-3 fatty acid supplementation for the secondary prevention of cardiovascular disease.

## Competing interests

The authors declare that they have no competing interests.

## Authors' contributions

JMB conceived of the study idea, and FE and JMB contributed to the study design. FE conducted the literature review. FE, MB, and KBF performed the data extraction and JMB, ND, and KBF were involved in consensus agreements concerning data discrepancies. FE and KBF drafted the manuscript. ND designed and ND and IS conducted the statistical analyses. All authors were involved in revising the article for important intellectual content, interpreting the data, and approved the final version to be published.

## Pre-publication history

The pre-publication history for this paper can be accessed here:

http://www.biomedcentral.com/1471-2261/10/24/prepub

## Supplementary Material

Additional file 1 **QUORUM Checklist**. Checklist detailing where the required elements of a thorough meta-analysis, as specified by the Quality of Reporting of Meta-Analyses (QUOROM) guidelines, can be located within the manuscript. Click here for file

Additional file 2 **Quality Assessment of Included Trials**. Quality assessment of randomized controlled trials examining the effect of omega-3 fatty acids on all-cause mortality and restenosis using the Jadad scale. Click here for file

Additional file 3 **Study and Patient Characteristics**. Study and patient characteristics of trials examining the effect of omega-3 fatty acids on all-cause mortality and coronary restenosis. Click here for file

Additional file 4 **Secondary Cardiovascular Outcomes**. Randomized controlled trials reporting secondary cardiovascular outcomes, including arrhythmias, cardiac death, congestive heart failure, implantable cardioverter defibrillator shocks, non-fatal myocardial infarction, stroke, and sudden death. Click here for file
