# Supplementary material for: Omega-3 fatty acids in high-risk cardiovascular patients: a meta-analysis of randomized controlled trials
Source: BMC Cardiovasc Disord. 2010 Jun 3;10:24. doi: 10.1186/1471-2261-10-24 (PMC2894745; doi:10.1186/1471-2261-10-24)
Supplement: Additional file 2 — Quality Assessment of Included Trials. Quality assessment of randomized controlled trials examining the effect of omega-3 fatty acids on all-cause mortality and restenosis using the Jadad scale. [file 1471-2261-10-24-S2.PDF]

Quality assessment of randomized controlled trials examining the effect of omega-3 fatty acids on all-cause mortality and restenosis using the Jadad scale [23].

Author	Randomization	Concealment of Allocation	Double Blinding	Description	Dropouts (intervention, control)	Total	Bias
Borchgrevink [24]	1	1	1	-1	0	2	High
Dehmer [31]	1	-1	0	0	1	1	High
Grigg [29]	1	1	1	-1	0	2	High
Milner [32]	1	1	0	0	1	3	Medium
Reis [30]	1	1	1	1	1	5	Low
Nye [39]	1	-1	1	1	1	3	Medium
Bairati [33]	1	1	1	-1	1	3	Medium
Bellamy [28]	1	-1	0	0	1	1	High
Kaul [27]	1	1	1	1	0	4	Low
Franzen [34]	1	1	1	1	1	5	Low
Leaf [25]	1	1	1	1	1	5	Low
Sacks [37]	1	-1	1	1	1	3	Medium
Cairns [26]	1	-1	0	0	1	1	High
Eritsland [40]	1	1	0	0	1	3	Medium
Rossing [41]	1	1	1	-1	1	3	Medium
Johansen [42]	1	-1	1	-1	1	1	High
von Schacky [36]	1	1	1	1	1	5	Low
Nilsen [43]	1	1	1	1	0	4	Low
Durrington [56]	1	1	1	0	1	4	Low
Marchioli [44]	1	1	0	0	0	2	High
Maresta [35]	1	1	1	1	1	5	Low
Burr [45]	1	1	0	0	1	3	Medium
Calò [46]	1	-1	0	0	1	1	High
Leaf [47]	1	1	1	1	0	4	Low
Raitt [48]	1	-1	1	-1	1	1	High
Brouwer [49]	1	1	1	1	1	5	Low
Yokoyama [50]	1	1	0	0	1	3	Medium
GISSI-HF [55]	1	1	1	1	1	5	Low
OMEGA-Trial [57]	1	1	1	0	1	4	Low
