# Supplementary material for: Omega-3 fatty acids in high-risk cardiovascular patients: a meta-analysis of randomized controlled trials
Source: BMC Cardiovasc Disord. 2010 Jun 3;10:24. doi: 10.1186/1471-2261-10-24 (PMC2894745; doi:10.1186/1471-2261-10-24)
Supplement: Additional file 3 — Study and Patient Characteristics. Study and patient characteristics of trials examining the effect of omega-3 fatty acids on all-cause mortality and coronary restenosis. [file 1471-2261-10-24-S3.PDF]

Study and patient characteristics of trials examining the effect of omega-3 fatty acids on all-cause mortality and coronary restenosis.

Author	Location	Year	Number of Patients (n)			Study Population	Age (Mean ± SD)		Women (%)	Previous MI (%)	Dosage (g/day)	Adherence (%)	Follow up (Months)	Drop Outs (n)		Drop Out (%)	Endpoint Reported (Mortality vs Restenosis)
			Total	Fish Oil	Control		Fish Oil	Control						Fish Oil	Control		
Borchgrevink [24]	Norway	1966	200	100	100	Male Patients with Recent or Impending MI	57.3	57.4	0	25	4.5g ALA	NR	10	NR	NR	NR	Mortality
Dehmer [31]	USA	1988	90	46	44	PCI Male Patients	56±8.9	56±9.6	0	66	5.4g EPA + DHA	NR	6-12	3	5	8.9	Mortality & Restenosis
Grigg [29]	Australia	1989	108	52	56	PCI Patients	51.8±9.9	55.1±11.6	18	NR	3g EPA + DHA	NR	3.3	NR	NR	NR	Mortality & Restenosis
Milner [32]	USA	1989	194	95	99	PCI Patients	59	59	28	NR	3.5g EPA+DHA	75	6	0	0	0	Restenosis
Reis [30]	USA	1989	222	124	62	PCI Patients	60±10	67±9	26	NR	6g EPA	65	6	22	10	17.2	Restenosis
Nye [39]	New Zealand	1990	108*	36 (Grp1)	37 (Grp2)	PCI Patients	54±8	55±8	29	NR	2.16g EPA	NR	10 – 12	0	0	0	Mortality
Bairati [33]	Canada	1992	205	107	98	PCI Patients	54±9	55±8	18	28	4.5 g EPA + DHA	93	6	48	38	42	Mortality & Restenosis
Bellamy[‡‡] [28]	UK	1992	120	60	53[‡‡]	PCI Patients	55	53	25	34	3g EPA+DHA	NR	6	0	7	5.8	Restenosis

Study and patients characteristics of trials examining the effect of omega-3 fatty acids on all-cause mortality and coronary restenosis (con't).

Author	Location	Year	Number of Patients (n)			Study Population	Age (Mean ± SD)		Women (%)	Previous MI (%)	Dosage (g/day)	Adherence (%)	Follow up (Months)	Drop Outs (n)		Drop Out (%)	Endpoint Reported (Mortality vs Restenosis)
			Total	Fish Oil	Control		Fish Oil	Control						Fish Oil	Control		
Kaul [27]	India	1992	107	58	49	PCI Patients	56±11	59±9	15	NR	3g EPA + DHA	NR	6	NR	NR	NR	Restenosis
Franzen [34]	Germany	1993	204	103	101	PCI Patients	57	56	18	42	3.15g EPA + DHA	NR	4	0	0	0	Mortality & Restenosis
Leaf [25]	USA	1994	551	275	276	PCI Patients	NR†	NR†	21	33	6.9g EPA +DHA	NR	6	69	69	25	Mortality & Restenosis
Sacks [37]	USA	1995	80	41	39	Patients with Documented CAD and Normal Lipids	62±7	62±7	7	56	6g (EPA + DHA + other n-3 FA	84	29	10‡	11‡	26	Mortality & Restenosis
Cairns [26]	Canada	1996	653	325	328	PCI Patients	57	56	18	50	5.4g of EPA + DHA	85	4.2	NR	NR	NR	Mortality & Restenosis
Eritsland [40]	Norway	1996	610	317	293	CABG Patients	60.5	61	13	52	3.3g EPA + DHA	88	12	15	14	4.6	Mortality
Rossing [41]	Denmark	1996	36	18	18	Normotensive Insulin Dependent Diabetics with Diabetic Nephhropathy	32±7	34±10	28	NR	4.6g EPA + DHA	NR	12	4‡	3‡	19	Mortality

Study and patients characteristics of trials examining the effect of omega-3 fatty acids on all-cause mortality and coronary restenosis (con't).

Author	Location	Year	Number of Patients (n)			Study Population	Age (Mean ± SD)		Women (%)	Previous MI (%)	Dosage (g/day)	Adherence (%)	Follow up (Months)	Drop Outs (n)		Drop Out (%)	Endpoint Reported (Mortality vs Restenosis)
			Total	Fish Oil	Control		Fish Oil	Control						Fish Oil	Control		
Johansen [42]	Norway	1999	500	250	250	PCI Patients	60.3±9.3	59.1±9.3	22	51	5.04g EPA + DHA	NR	6	54	58	22.4	Mortality & Restenosis
von Schacky [36]	Germany	1999	223	112	111	Patients with Angio-graphically-proven CAD	57.8±10	58.9±8	20	52	6g EPA + DHA X 3 mo then 3g/day	93	24	NR	NR	NR	Mortality & Restenosis
Nilsen [43]	Norway	2001	300	150	150	Patients with Acute MI	64.4	63.6	21	23	3.5 g EPA and DHA	84	24	NR	NR	NR	Mortality
Durrington [56]	UK	2001	59	30	29	Patients with CAD and Persisting Hypertriglyceridemia	55.2±7.0	54.8±10.2	27	NR	4g/day EPA + DHA	95	6	1	1	96.6	Mortality
Marchioli [44]	Italy	2002	11,323	5,665	5,658	Patients with MI <3 Months	59.3	59.5	15	12	0.9g EPA and DHA	73	42	7	6	0.1	Mortality
Maresta [35]	Italy	2002	339	169	170	PCI Patients	58.9±9.5	58.6±8.7	16	48	5.1g EPA +DHA[¶]	86	≤ 6 post-PCI	44	38	24.2	Mortality & Restenosis
Burr [45]	UK	2003	3,114	1,571	1,543	Males Patients with Angina	61±6.5	61±6.3	0	50	3g	NR	36-108	0	0	0	Mortality
Calò [46]	Italy	2005	160	79	81	CABG Patients	66.2±8.0	64.9±9.1	15	53	1.75g (EPA + DHA)	98	1	0	0	0	Mortality
Leaf [47]	Norway	2005	402	200	202	ICD Patients	65.7±11.6	65.3±11.7	17	NR	2.6g EPA + DHA	64	12	NR	NR	NR	Mortality

Study and patients characteristics of trials examining the effect of omega-3 fatty acids on all-cause mortality and coronary restenosis (con't).

Author	Location	Year	Number of Patients (n)			Study Population	Age (Mean ± SD)		Women (%)	Previous MI (%)	Dosage (g/day)	Adherence (%)	Follow up (Months)	Drop Outs (n)		Drop Out (%)	Primary Endpoint Reported
			Total	Fish Oil	Control		Fish Oil	Control						Fish Oil	Control		
Raitt [48]	USA	2005	200	100	100	Patients with ICDs and Prior Ventricular Fibrillation or Tachycardia	63±13	62±13	14	56	1.8g EPA + DHA	79	24 (Median)	2	6	4	Mortality
Brouwer [49]	8 European countries	2006	546	273	273	Patients with ICDs and Prior Ventricular Fibrillation or Tachycardia	60.5±12.8	62.4±11.4	16	63	2g EPA + DHA + other omega3	65	12	29	25	9.9	Mortality
Yokoyama[‖] [50]	Japan	2007	3,664	1,823	1,841	Secondary prevention	61±9	61±8	68	6	1.8g/d EPA	72	55	99	98	5.4	Mortality
GISSI-HF[††] [55]	Italy	2008	6,975	3,494	3,481	CHF Patients with New York Heart Association class II-IV	67±11	67±11	22	41	1g (EPA + DHA)	72	47 (Median)	37	46	1.2	Mortality
OMEGA-Trial [57]	Germany	2009	3,851	1,940	1,911	Post-MI Patients	64	64	26	100	1g/day	NR	12	21	26	1.2	Mortality

Abbreviations: ALA: α linolenic acid; CABG: coronary artery bypass graft surgery; CAD: coronary heart disease; CHF: congestive heart failure; DHA: docosahexaenoic acid; EPA: eicosapentaenoic acid; FA: omega-3 fatty acid; ICD: implantable cardioverter defibrillator; MI: myocardial infarction; NR: not reported; PCI: percutaneous coronary intervention;

* Randomly divided into 3 groups: Group 1:EPA, Group 2: Placebo, and Group 3: ASA/Dypirimidole.

† Age data were categorized. In the fish oil group, 4% were 30-39 years old, 19% were 40-49 years old, 27% were 50-59 years old, 38% were 60-69 years old, and 12% were ≥70 years old. In the corn oil group, 4% were 30-39 years old, 20% were 40-49 years old, 27% were 50-59 years old, 38% were 60-69 years old, and 11% were ≥70 years old.

‡ All patients who dropped out were monitored for outcomes.

¶ Patients were randomized to 5.1g/day of EPA +DHA for the first 2 months and half dose for the next 6 months.

‖ The JELIS Trial (conducted by Yokoyama) [50] was performed on a population with low, moderate and high risk cardiovascular patients. Due to the lack of data, cardiovascular instead of all-cause mortality was used for the secondary prevention group. Demographic and cardiovascular outcome data are presented for the high-risk group (n=3,664) whereas safety data are presented for the entire study population (n=18,645). The JELIS cardiovascular data were available for the secondary prevention group and are included as part of our pooled analysis. However, due to the heterogeneity of the entire study population, data from the JELIS trial were not included in our pooled analysis of safety data as separate safety data were not reported for the secondary prevention sub-cohort. Age, women, MI, and drop outs are reported for the whole JELIS study and do not refer to the secondary prevention sub-cohort.

[††] In the GISSI-HF study [55], 7,046 patients were randomized but only 6,975 patients were included in the analyses. The remaining 71 patients were excluded prior to unblinding due to data quality concerns. Although these patients should be included in the intention-to-treat analyses, these data were not available and thus excluded from our analysis.
[‡‡] The control group involved a total of 60 patients, 53 of which underwent follow-up angiography and 7 of which who did not. Consequently, the sample size of the control group is 53 patients for baseline and restenosis data but 60 patients for all other analyses.
