# Supplementary material for: Omega-3 fatty acids in high-risk cardiovascular patients: a meta-analysis of randomized controlled trials
Source: BMC Cardiovasc Disord. 2010 Jun 3;10:24. doi: 10.1186/1471-2261-10-24 (PMC2894745; doi:10.1186/1471-2261-10-24)
Supplement: Additional file 4 — Secondary Cardiovascular Outcomes. Randomized controlled trials reporting secondary cardiovascular outcomes, including arrhythmias, cardiac death, congestive heart failure, implantable cardioverter defibrillator shocks, non-fatal myocardial infarction, stroke, and sudden death. [file 1471-2261-10-24-S4.PDF]

Randomized controlled trials reporting secondary cardiovascular outcomes.

	Number of Patients		Sudden Death		Cardiac Death		Non-Fatal MI		CHF		Arrhythmias		ICD Shock		Stroke	
Study	Fish Oil	Ctrl	Fish Oil	Ctrl	Fish Oil	Ctrl	Fish Oil	Ctrl	Fish Oil	Ctrl	Fish Oil	Ctrl	Fish Oil	Ctrl	Fish Oil	Ctrl
Borchgrevink [24]	100	100	3	4	10	12	10	4	NR	NR	NR	NR	NR	NR	0	1
Dehmer [31]	43	39	NR	NR	NR	NR	1	0	NR	NR	NR	NR	NR	NR	NR	NR
Grigg [29]	52	56	1	0	1	0	1	0	NR	NR	NR	NR	NR	NR	NR	NR
Milner [32]	95	99	0	0	0	0	NR	NR	NR	NR	NR	NR	NR	NR	NR	NR
Reis [30][*]	124[*]	51[*]	0	0	0	0	7	0	NR	NR	NR	NR	NR	NR	NR	NR
Nye [39]	36	37	0	0	0	0	NR	NR	NR	NR	NR	NR	NR	NR	NR	NR
Bairati [33]	59	60	0	0	0	0	NR	NR	NR	NR	NR	NR	NR	NR	NR	NR
Bellamy [28][†]	60	60[†]	0	0	0	0	NR	NR	NR	NR	NR	NR	NR	NR	NR	NR
Kaul [27]	58	49	NR	NR	NR	NR	4	2	NR	NR	NR	NR	NR	NR	NR	NR
Franzen [34] [‡]	103[‡]	101[‡]	1	2	NR	NR	NR	NR	NR	NR	NR	NR	NR	NR	NR	NR
Leaf [25]	226	221	0	1	0	2	NR	NR	NR	NR	NR	NR	NR	NR	NR	NR
Sacks [37] [§]	41[§]	39[§]	NR	NR	0	1	1	2	0	1	0	0	NR	NR	1	0
Cairns [26]	325	328	NR	NR	NR	NR	3	5	NR	NR	NR	NR	NR	NR	0	0
Eritsland [40]	317	293	7	4	NR	NR	NR	NR	NR	NR	NR	NR	NR	NR	NR	NR
Rossing [41]	18	18	0	0	0	0	0	0	0	0	0	0	NR	NR	0	0
Johansen [42][¶]	196[¶]	192[¶]	NR	NR	1	3	NR	NR	NR	NR	NR	NR	NR	NR	NR	NR
von Schacky [36]	111	112	0	0	0	1	1	3	0	0	0	0	NR	NR	1	3
Nilsen [43]	150	150	NR	NR	8	8	21	15	NR	NR	NR	NR	NR	NR	NR	NR
Durrington [56]	30	29	0	0	0	1	0	0	0	0	0	0	NR	NR	0	0
Marchioli [44]	5,666	5,658	111	154	247	306	223	233	NR	NR	NR	NR	NR	NR	92	77
Maresta [35] [‖]	146[‖]	141[‖]	NR	NR	NR	NR	1	0	NR	NR	NR	NR	NR	NR	NR	NR
Burr [45]	1571	1543	73	47	180	139	NR	NR	NR	NR	NR	NR	NR	NR	NR	NR
Calò [46]	79	81	NR	NR	NR	NR	NR	NR	NR	NR	12	27	NR	NR	NR	NR
Leaf [47][**]	200	202	3[**]	1[**]	9	9	NR	NR	NR	NR	NR	NR	57	78	NR	NR
Raitt [48]	100	100	2	0	2	5	1	3	14	12	21	16	65	59	NR	NR
Brouwer [49]	273	273	NR	NR	6	13	1	3	22	19	36	34	75	81	NR	NR
Yokoyama [50][††]	1,823	1,841	13	13	18	21	26	38	NR	NR	NR	NR	NR	NR	NR	NR
GISSI-HF [55][‡‡]	3,494	3,481	307	325	712	765	87	104	978	995	274[‡‡]	304[‡‡]	NR	NR	112	103
OMEGA-Trial [57]	1,919	1,885	29	28	67	51	86	77	466	492	21	13	10[***]	2[***]	27	13
**Relative Risk (95% CrI)**			**0.90 (0.64, 1.15)**		**0.84 (0.57, 1.03)**		**0.91 (0.72, 1.11)**		**0.95 (0.67, 1.18)**		**0.88 (0.49, 1.30)**		**0.92 (0.43, 1.91)**		**1.04 (0.43, 1.56)**	

Abbreviations: CHF=congestive heart failure; ICD= implantable cardioverter defibrillator; MI=myocardial infarction; NR=not reported.

* Reis et al. [30] randomized 222 patients, 204 of which underwent angioplasty. Of these patients, 14 patients had unsuccessful angioplasties and 4 other patients underwent bypass surgery following their angioplasty. These 18 patients were excluded from further study. The remaining 186 patients were included in the study, including 124 patients in the fish oil group (60 randomized to ethyl ester fish oil, 64 randomized to triglyceride fish oil) and 62 randomized to placebo. Clinical event data were available for all patients however 11 patients in the placebo group did not provide side effect data. Thus, side effect analyses are based on 124 fish oil patients and 51 placebo patients.

† The control group involved a total of 60 patients, 53 of which underwent follow-up angiography and 7 of which who did not. Consequently, the sample size of the control group is 53 patients for baseline and restenosis data but 60 patients for all other analyses.

‡ In the study by Franzen and colleagues [34], 204 patients were randomized to fish oil (n=103) or placebo (n=101). However, only 175 patients underwent angiographic follow-up (92 fish oil and 83 placebo). Consequently, the sample size for restenosis is 175 patients whereas the sample size for all other analyses is 204.

§ Sacks and colleagues [37] randomized 80 patients to fish oil (n=41) or control (n=39). A total of 21 patients were excluded from the study prior to undergoing their follow-up angiogram. Although clinical events were assessed in all 80 randomized patients, restenosis was only assessed in 59 patients (31 fish oil, 28 control).

¶ Johansen and colleagues [42] randomized a total of 500 patients to omega-3 fatty acid (n=250) or placebo (n=250). Of these patients, 5 were disqualified before their angioplasty (4 omega-3 patients and 1 placebo patient), 103 were disqualified during their angioplasty (n=49 and 54, respectively), and 4 patients were excluded because they died prior to their follow-up angiogram (1 omega-3 patient and 3 patients randomized to placebo). There were therefore 196 patients randomized to omega-3 fatty acids and 192 patients randomized to placebo who were ultimately included in the study. The cardiac death analyses are based on 197 patients randomized to omega-3 fatty acids and 195 patients randomized to placebo whereas all other analyses are based on 196 and 102 patients, respectively.

‖ Maresta et al. [35] randomized 339 patients (169 to fish oil, 170 to placebo). Of these patients, 52 were disqualified before or at their angioplasty, leaving 287 patients (146 fish oil, 141 placebo). Side effect and clinical event data are based on these patients. An additional 23 patients were unevaluable drop-outs, leaving 264 patients (129 fish oil, 135 placebo) with 6-month angiograms available, and 7 additional patients were excluded because quantitative coronary angiography was not possible, resulting in a total of 257 patients (125 fish oil, 132 placebo) being included in the restenosis analyses.

** Leaf and colleagues [47] reported arrhythmic deaths, which we have considered as sudden deaths.

†† The JELIS Trial (conducted by Yokoyama) [50] was performed on a population with low, moderate and high risk cardiovascular patients. Due to the lack of data, cardiovascular instead of all-cause mortality was used for the secondary prevention group. Demographic and cardiovascular outcome data are presented for the high-risk group (n=3,664) whereas safety data are presented for the entire study population (n=18,645). The JELIS cardiovascular data were included as part of our pooled analysis. However, due to the heterogeneity of the entire study population, data from the JELIS trial were not included in our pooled analysis of safety data.

‡‡ In the GISSI-HF study [55], 7,046 patients were randomized but only 6,975 patients were included in the analyses. The remaining 71 patients were excluded prior to unblinding due to data quality concerns. Although these patients should be included as part of the intention-to-treat analyses, these data were not available and thus excluded from our analysis. In addition, the GISSI-HF investigators reported presumed arrhythmic deaths rather than arrhythmic events.

*** In the OMEGA Trial [57], not all patients received ICDs. Consequently, not all patients were eligible for this outcome, and this study was therefore excluded from our analysis of ICD shocks.
